# Letter to the editor: Labral calcification plays a key role in hip pain and symptoms in femoroacetabular impingement

**DOI:** 10.1186/s13018-020-01744-0

**Published:** 2020-06-12

**Authors:** Mingjin Zhong, Kan Ouyang, Wei Lu

**Affiliations:** 1 Department of Sports Medicine, The First Affiliated Hospital of Shenzhen University, Health Science Center, Shenzhen, China; 2 The Second People’s Hospital of Shenzhen, No.3002 Sungang West Road, Futian district, Shenzhen, 518000 Guangdong province China

To the Editor:

Recently, we have read the research article entitled “Labral calcification plays a key role in hip pain and symptoms in femoroacetabular impingement” by Trisolino G and his colleagues [1]. The authors investigated the joint tissue status at the time of arthroscopic treatment for FAI and found a new insight into the relationship between femoroacetabular impingement (FAI) and hip osteoarthritis (OA). We really appreciate the work that has been done by the authors. However, we would also like to point some of our concerns regarding the paper.

First, the authors did not collect normal labrum samples from healthy hip joints. We have known that calcification deposition could also be found in healthy labral tissues [2]. Thus, labral calcification may not just be considered as a result or byproduct of impingement and degeneration, but rather seems a normal physiological process.

Second, FAI involved the abutment of the femoral head against the acetabulum in a hip flexed-adducted-internally rotated position, impinging the anterior–superior portion of the labrum, and resulting in varied and limited labrum lesion [3]. Accordingly, we speculate that the intraoperative collected labrum specimens reflect the limited anterosuperior region, but in absolute terms, only a small part of the acetabular labrum. However, OA is a whole-joint disorder involving the whole part of the acetabular labrum. Therefore, only the anterior–superior part of each labrum from end-stage OA patients should be collected in order to avoid selection bias.

Third, several studies have characterized the calcification of soft tissues in the end-stage OA hip joints [2, 4]. To our knowledge, the calcified hyaline articular cartilage contained basic calcium phosphate (BCP) and calcium pyrophosphate dihydrate (CPPD) crystals; however, only CPPD and no BCP crystals deposited in the acetabular labrum [2]. It is not clear whether BCP crystals could be found in labrum calcification of FAI patients. We do think the pathophysiologic characteristics of the labrum calcification studied do not appear to be from degenerative changes, but a separate pathophysiologic process from damage to the labrum from FAI leading to a calcific response [5]. This needs to be confirmed in future studies.

Moreover, the authors concluded that a higher local concentration of calcium crystal deposition in the labrum could lead to a higher pain level and worse functional sore. Previous study stated that the amount of calcification in the labrum instead of histological degeneration grade had a significant influence on the preoperative Harris Hip Score (HHS) in patients with end-stage OA [2, 4]. Therefore, how much amount of calcium crystal deposition in the labrum produce adverse effect on hip function in patients with FAI need further study.

## Data Availability

Not applicable.

## Abbreviations

BCP: Basic calcium phosphate
CPPD: Calcium pyrophosphate dihydrate
FAI: Femoroacetabular impingement
HHS: Harris Hip Score
OA: Osteoarthritis
